# Natural products alleviate viral pneumonia by modulating inflammatory and oxidative-stress pathways

**DOI:** 10.3389/fphar.2025.1657829

**Published:** 2025-08-11

**Authors:** Yifu Tie, Han Liu, Tong Zhang, Tianwei Meng, Qun Liang

**Affiliations:** ^1^ Heilongjiang University of Chinese Medicine, Harbin, China; ^2^ Ordos Hospital of Traditional Chinese Medicine, Ordos, China; ^3^ Institute for Global Health, University College London, London, United Kingdom; ^4^ The First Affiliated Hospital, Heilongjiang University of Chinese Medicine, Harbin, China

**Keywords:** viral pneumonia, natural products, inflammatory factor, oxidative stress, pathological mechanism

## Abstract

Viral pneumonia, primarily caused by influenza viruses, coronaviruses, and other respiratory pathogens, is characterized by direct alveolar epithelial injury and an excessive immune response, leading to severe inflammation, oxidative stress, and, in critical cases, acute respiratory distress syndrome and multi-organ failure. Traditional Chinese Medicine (TCM), widely employed in China for both the prevention and treatment of viral pneumonia, provides multitarget and broad-spectrum therapeutic benefits with low toxicity and minimal side effects, offering a promising alternative to conventional antiviral therapies. Recent studies have demonstrated that natural products derived from TCM, including flavonoids, polyphenols, polysaccharides, and terpenoids, can effectively modulate immune and oxidative stress responses by targeting multiple signaling pathways. In this review, we conducted a systematic literature search in PubMed, Web of Science, and SciFinder databases, focusing primarily on studies published over the past decade. Keyword combinations included “viral pneumonia,” “Traditional Chinese Medicine,” “natural products,” “inflammation,” and “oxidative stress,” in addition to mechanism-related terms such as “NF-κB,” “Nrf2,” “PI3K/Akt,” “MAPK,” and “NLRP3 inflammasome.” Natural compounds acting on these pathways have been shown to suppress cytokine storms, reduce reactive oxygen species accumulation, preserve alveolar epithelial integrity, and alleviate pulmonary inflammation. This review highlights the latest progress in understanding how natural products exert protective effects in viral pneumonia through the modulation of inflammation and oxidative stress–related pathways. These findings provide a theoretical foundation for developing novel anti-inflammatory and antioxidant therapeutic strategies based on natural medicines for the treatment of viral respiratory diseases.

## 1 Introduction

Viral pneumonia—principally caused by influenza viruses, coronaviruses, and related pathogens—is an inflammatory condition of the lung in which direct viral injury to the alveolar epithelium, coupled with an exaggerated host immune response, leads to impaired gas exchange and, in severe cases, multi-organ failure (Figueiredo, 2009; Ruuskanen et al., 2011; Febbo et al., 2024). As the fundamental pathological event underlying influenza, Coronavirus Disease 2019(COVID-19), and other major respiratory illnesses, viral pneumonia is the primary cause of mortality in acute respiratory infections and predisposes survivors to secondary bacterial infections or chronic pulmonary fibrosis, thereby increasing clinical complexity (Lee et al., 2016; Rasool et al., 2024). The latest data indicate that viruses are responsible for exceeding 30% of community-acquired pneumonia cases worldwide (Ruuskanen et al., 2011; Jain et al., 2015). During the COVID-19 pandemic, the mortality rate among severely ill patients exceeded 10% at one point, causing a profound impact on healthcare systems and the economy (Richardson et al., 2020; Zhou et al., 2020). In the face of continual viral mutation and immune evasion, combined anti-inflammatory and antioxidant therapy has become crucial for improving outcomes (Cecchini and Cecchini, 2020; Suhail et al., 2020). The development of precise immunomodulatory and tissue-repair strategies constitutes an urgent research priority.

**FIGURE 1:**
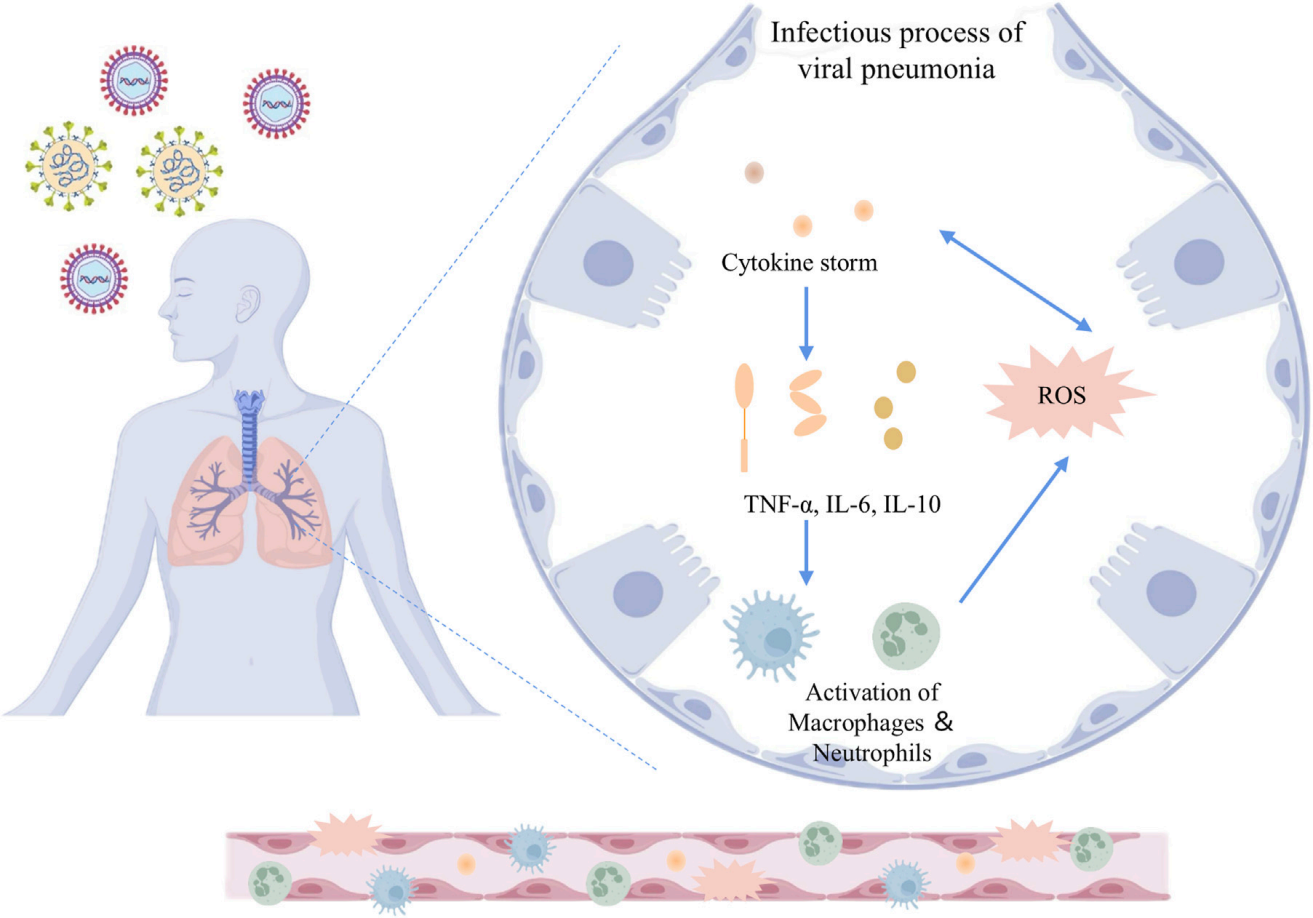
Inflammatory and oxidative stress mechanisms in the infectious process of viral pneumonia. The schematic illustrates the pathophysiological response to viral infection in the lungs. Upon viral invasion, the host immune system triggers a cytokine storm characterized by elevated levels of pro-inflammatory mediators such as TNF-α, IL-6, and IL-10. This cytokine cascade promotes the activation of macrophages and neutrophils, which further amplifies the inflammatory response. Concurrently, the production of ROS is upregulated, contributing to oxidative stress and cellular damage. The release of cytokines and the generation of ROS mutually reinforce each other, constituting one of the key mechanisms in the pathogenesis of viral pneumonia. TNF-α: Tumor necrosis factor-alpha; IL-6: Interleukin-6; IL-10: Interleukin-10; ROS: Reactive oxygen species.

**FIGURE 2:**
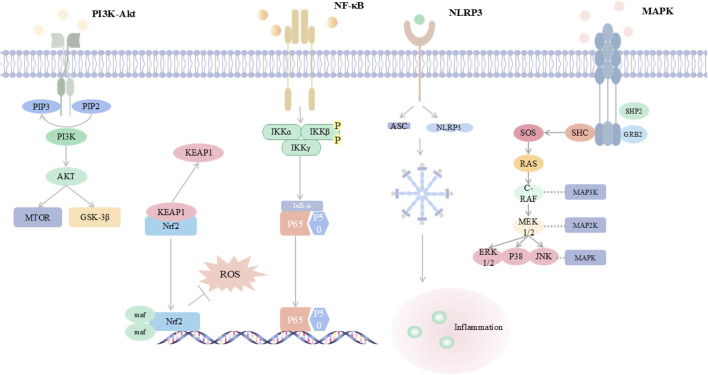
Integrated Map of Core Inflammation-Oxidative Stress Signalling Networks This schematic summarizes five key pathways in viral pneumonia: PI3K/Akt signals through mTOR and GSK-3β and modulates Nrf2-mediated antioxidant defense; NF-κB is activated through IKK to induce pro-inflammatory cytokines; the NLRP3 inflammasome assembles with ASC and triggers inflammation; MAPK cascades include ERK, p38, and JUN and amplify cytokine production. A cytokine–ROS positive feedback drives oxidative stress, inflammation, and lung injury. Abbreviations are provided in the table in the main text.

Inflammation and oxidative stress are the core pathological features of viral pneumonia: after viral invasion, on the one hand, it activates immune cells (such as macrophages and neutrophils) to release pro-inflammatory factors, such as interleukin-6(IL-6) and tumor necrosis factor-alpha (TNF-α), triggering a “cytokine storm”, leading to the destruction of the alveolar-capillary barrier and pulmonary edema; on the other hand, viral replication and inflammatory responses induce excessive production of reactive oxygen species (ROS) and reactive nitrogen species (RNS), causing lipid peroxidation, deoxyribonucleic acid (DNA) damage and mitochondrial dysfunction, further aggravating tissue oxidative damage (Cecchini and Cecchini, 2020; Merad and Martin, 2020; Suhail et al., 2020; Ye et al., 2020). The two form a vicious cycle through mutual amplification: inflammatory signals drive ROS production, while oxidative stress activates pathways such as the NOD-like receptor family pyrin domain-containing 3 (NLRP3) inflammasome, promoting the spread of inflammation (Suhail et al., 2020). This synergistic effect not only intensifies alveolar epithelial cell apoptosis and pulmonary interstitial fibrosis but also triggers systemic multi-organ damage and failure, constituting the key mechanism for the high mortality rate of viral pneumonia. The inflammatory and oxidative stress responses involved in the development of viral pneumonia are related to abnormalities in several signaling pathways. Viral infection activates inflammatory pathways through pattern recognition receptors, leading to atypical high expression of pro-inflammatory factors, while the inflammatory cascade exacerbates oxidative stress and mitochondrial dysfunction (Merad and Martin, 2020). The bidirectional vicious cycle of “inflammation-oxidative stress” becomes the pathogenesis of viral pneumonia (Figure 1). This article reviews the nuclear factor kappa-light-chain-enhancer of activated B cells (NF-κB) signaling pathway, phosphoinositide 3-kinase/protein kinase B (PI3K/Akt) signaling pathway, nuclear factor erythroid 2-related factor 2 (Nrf2) signaling pathway, mitogen-activated protein kinase (MAPK) signaling pathway, NLRP3 inflammasome signaling pathway, etc., involved in the pathological process of viral pneumonia. It emphasizes the core molecular structures of the above pathways, their dynamic interactions, and their key roles in alveolar epithelial apoptosis, immune imbalance, and pulmonary fibrosis, providing a theoretical basis and new therapeutic strategies for targeted inhibition of the inflammatory storm and restoration of redox homeostasis (Figure 2).

**FIGURE 3:**
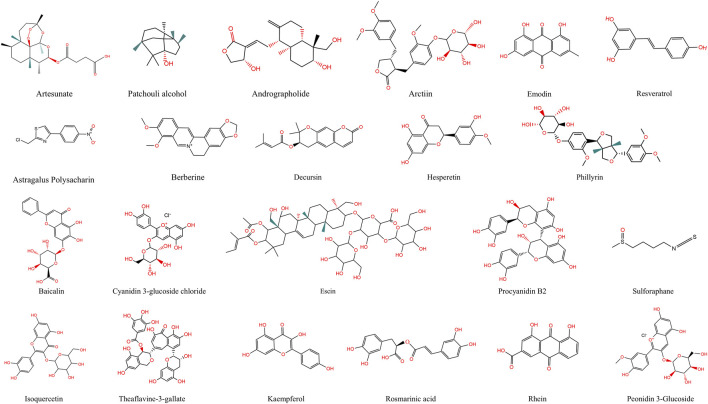
Chemical Structures of Natural Products. This figure shows the chemical structures of 22 active compounds derived from natural sources.

The most widely used Western medicines for viral pneumonia are oseltamivir and remdesivir. Oseltamivir inhibits influenza neuraminidase and thus blocks viral release from host cells, whereas remdesivir, a ribonucleic acid (RNA)-dependent RNA-polymerase inhibitor, interferes with the replication of coronaviruses and other RNA viruses (Thorlund et al., 2011). Prolonged use of these drugs, however, is limited by high cost, emerging resistance, and adverse effects (notably gastrointestinal reactions and hepato-renal dysfunction), highlighting the need for safer and more economical alternatives (Thorlund et al., 2011). Traditional Chinese Medicine (TCM), with its multicomponent, multitarget characteristics, offers unique advantages in modulating inflammation and oxidative stress. Active ingredients such as baicalin and quercetin attenuate lung injury by suppressing NF-κB and NLRP3 inflammasome activation while simultaneously inducing Nrf2-mediated antioxidant defences with relatively low toxicity (Sul and Ra, 2021; Shen et al., 2023; Wen et al., 2023). During the COVID-19 pandemic, several clinical studies have confirmed the therapeutic potential of TCM formulations. For example, a multicenter randomized controlled trial showed that Lianhua Qingwen significantly shortened the duration of fever, cough, and fatigue in patients with mild to moderate COVID-19, while reducing radiographic lung lesion progression compared to control treatment (Zhan et al., 2023). Similarly, Xuebijing injection, in a real-world cohort study, was associated with improved oxygenation index, reduced IL-6 levels, and lower mortality in severe COVID-19 cases (Luo et al., 2020). These findings highlight the potential of TCM as an adjunct to conventional therapy in managing viral pneumonia. Nevertheless, the complex chemical composition of herbal formulae and their multi-pathway actions remain incompletely characterised, and systematic investigation of individual natural products is still inadequate. To address this, we conducted a systematic literature search using PubMed, Web of Science, and SciFinder, focusing primarily on studies published within the past 10 years. Additionally, a few earlier seminal studies were included due to their foundational contributions to the understanding of compound–target–pathway interactions in viral pneumonia. The search strategy employed combinations of keywords including “viral pneumonia,” “traditional Chinese medicine,” “natural product,” “inflammation,” and “oxidative stress,” along with mechanism-related terms such as “NF-κB,” “Nrf2,” “PI3K/Akt,” “MAPK,” and “NLRP3 inflammasome” to ensure accurate and relevant retrieval. Based on this systematic review of the literature, this article summarizes current advances in the modulation of inflammation–oxidative stress networks by natural products and elucidates their compound–target–pathway interactions, thus providing theoretical foundations and innovative therapeutic perspectives for the treatment of viral pneumonia (Table 1; Figure 3).

**TABLE 1:** Summary of *in vivo* and *in vitro* studies on representative natural compounds: experimental design, dosing, and mechanistic indicators.

Active ingredient	Plants	Chemical class	Study type	Experimental model	Sex	Dose range tested	Minimal active concentration	Positive drug group	Duration	Detection indicators	Signaling pathways	Ref
Artesunate	*Artemisia annua*	Sesquiterpene lactone	*In Vivo*	ICR mice	NR	30, 60, 120 mg/kg/day	60 mg/kg/day	Oseltamivir	7 d	TLR4,NF-κB,TNF-α,IL-6,IL-1β	NF-κB	Zhou et al. (2022)
Patchouli alcohol	*Pogostemon cablin Benth*	Sesquiterpene	*In Vivo*	BALB/c mice	Female	10, 20, 40 mg/kg/day	10 mg/kg/day	Oseltamivir	5 d	IL-1β, IL-6, TNF-α, NLRP3, Caspase-1, GSDMD, ASC, NF-κB, USP1	NF-κB, NLRP3	Jiang et al. (2025)
			*In Vitro*	MDCK cell, A549 cell	NA	6, 12, 24 μM	6 μM	Oseltamivir	12–24 h	IL-1β, IL-6, TNF-α, p-IKKβ, p-IκBα, p-NF-κB p65, NLRP3, Caspase-1, GSDMD, ASC	NF-κB, NLRP3	Jiang et al. (2025)
			*In Vivo*	Kunming mice	Female	1.56–100 μg/mL	2.2 μg/mL	Oseltamivir phosphate	4–7 d	IFN-γ, IL-2, p-PI3K, p-Akt	PI3K/Akt, MAPK	Yu et al. (2019)
			*In Vitro*	MDCK cell, A549 cell	NA	0.25–250 μg/mL	25–50 μg/mL	Oseltamivir carboxylate/Zanamivir/Nucleozin	24 h	p-PI3K, p-Akt, p-ERK1/2, p-NF-κB, IFN-β	PI3K/Akt, MAPK	Yu et al. (2019)
Andrographolide	*Andrographis paniculata*	Diterpenoid lactone	*In Vivo*	C57BL/6 mice	Female	10 mg/kg/day	10 mg/kg/day	Oseltamivir	7 d	IL-6, IL-10, TNF-α, IFN-γ, TGF-β,p-NF-κB,STAT1, STAT2, p-STAT1, p-STAT2	NF-κB, JAK-STAT	Li et al. (2017)
			*In Vitro*	MDCK, THP-1, Raw 264.7, A549 cell	NA	10–50 μM	10 μM	Oseltamivir	24 h	IL-6, TNF-α, IFN-γ, IL-10, TGF-β,STAT1, STAT2, p-STAT1, p-STAT2, IRF9	NF-κB, JAK-STAT	Li et al. (2017)
Isoquercitrin	*Houttuynia cordata*	Flavonoid	*In Vivo*	C57BL/6 mice	Male	10 mg/kg/day	10 mg/kg/day	Dexamethasone	5 d	IFN-α, IFN-β, TNF-α, IL-1β, IL-6, IL-10, ISG54, ISG56, IκBα, p-IκBα, p65, p-p65	NF-κB, JAK-STAT	Luo et al. (2023)
			*In Vitro*	MDCK cell and A549 cell	NA	8–250 μg/mL	42.63–48.90 μg/mL	Dexamethasone	48 h	IFN-α, IFN-β, TNF-α, IL-1β, IL-6, IL-10, ISG54, ISG56, IκBα, p-IκBα, p65, p-p65	NF-κB, JAK-STAT	Luo et al. (2023)
β-Escin	*Aesculus hippocastanum*	Saponin	*In Vitro*	Vero E6, Calu-3, CRFK epithelial cell, J774A.1 macrophage	NA	1–30 μg/mL	1.3 μg/mL	NA	26 h	IL-6, TNF-α, NF-κB	NF-κB	Peñaranda Figueredo et al. (2024)
Phillyrin	*Forsythia suspensa leaf*	Lignan	*In Vivo*	BALB/c mice	Female	50, 100, 200 mg/kg/day	100 mg/kg/day	Ribavirin	4 d	IL-6, IL-1β, PI3K, Akt, Nrf2, NLRP3, IL-18, β-actin	PI3K/Akt-Nrf2, NLRP3	Zhang et al. (2023)
Rosmarinic acid	*Forsythia suspensa leaf*	Phenolic acid	*In Vivo*	BALB/c mice	Female	50, 100, 200 mg/kg/day	100 mg/kg/day	Ribavirin	4 d	IL-6, IL-1β, PI3K, Akt, Nrf2, NLRP3, IL-18, β-actin	PI3K/Akt-Nrf2, NLRP3	Wang et al. (2024)
Resveratrol	*Polygonum cuspidatum*	Stilbenoid	*In Vivo*	BALB/c mice	Male and female	10–100 mg/kg/day	30–50 mg/kg/day	Wortmannin	7 d	IL-6, TNF-α, PI3K, p-Akt, Caspase-3	PI3K/Akt	Li et al. (2020)
Decursin	*Angelica decursiva*	Coumarin	*In Vivo*	C57BL/6 mice	Male and female	50, 100 mg/kg/day	50 mg/kg/day	oseltamivir phosphate	3 d	IL-6, L-17, TNF-α, IL-10, PI3K, p-PI3K, AKT, p-AKT	PI3K/Akt	Tang et al. (2025)
Andrographolide	*Andrographis paniculata*	Diterpenoid lactone	*In Vivo*	BALB/c mice	Male and female	20 mg/kg/day	20 mg/kg/day	Remdesivir	5 d	IL-6, IL-10, TNF-α, IL-1β, NRF2, PI3K, AKT, p-AKT, GCLC	Nrf2	Chaopreecha et al. (2025)
			*In Vitro*	Calu-3 cell, Vero E6 cell, PHAE cell	NA	1.25–10 µM	1.25 µM	Remdesivir	48 h	NRF2, GCLC	Nrf2	Chaopreecha et al. (2025)
R-Sulforaphane	*Brassica oleracea* var. *italica*	Isothiocyanate	*In Vivo*	ICR mice	Male	9 μmoL/100 μL	9 μmoL/100 μL	NA	7 d	IL-6, IL-10, IL-13, IL-18, IFN-γ, Nrf2, GCLc, HO-1, NQO1, GST-P1, GPx2, Muc5ac, β-actin	Nrf2, NF-κB	Cho et al. (2009)
Berberine	*Coptis chinensis*	Isoquinoline alkaloid	*In Vivo*	BALB/c mice	Male	30, 45, 60 mg/kg/day	30 mg/kg/day	Ribavirin	4 d	IL-6, TNF-α, Nrf2, Gpx4	Nrf2	Zhang et al. (2016)
			*In Vitro*	J774A.1cell	NA	4.2, 8.4, 16.8 μM	4.2 μM	MCC950	24 h	IL-1β, TNF-α, LDH, GSDMD-N, NLRP3, ASC, Caspase-1, MAVS	NLRP3	Zhao et al. (2025)
Emodin	*Rheum palmatum*	Anthraquinone	*In Vivo*	C57BL/6J mice	Male and female	25–75 mg/kg/day	25 mg/kg/day	Oseltamivir	6 d	TNF-α	Nrf2, p38/JNK MAPK, NF-κB	Dai et al. (2017)
			*In Vitro*	MDCK cell, A549 cell	NA	6.25–25 μg/mL	6.25 μg/mL	Ribavirin	48 h	TLR, MAPK, NF-κB, Nrf2, IL-6, IL-8, TNF-α, IL-1β, IFN-γ, ROS, SOD, GR, CAT, GPx	Nrf2, p38/JNK MAPK, NF-κB	Dai et al. (2017)
			*In Vivo*	BALB/c mice	Female	20 mg/kg/day	20 mg/kg/day	Ribavirin	5 d	IL-1β, IL-18, TNF-α, IL-33, NLRP3, ASC, Caspase-1, NF-κB, p-NF-κB	NF-κB, NLRP3	Shen et al. (2019)
			*In Vitro*	BEAS-2B cell	NA	5, 10, 20 μM	10 μM	Ribavirin	24 h	IL-1β, IL-18, TNF-α, IL-6, NLRP3, ASC, Caspase-1, NF-κB, p-NF-κB	NF-κB, NLRP3	Shen et al. (2019)
Kaempferol	*Radix Bupleuri*	Flavonoid	*In Vivo*	C57BL/6J mice	Male	80 mg/kg/day	80 mg/kg/day	NA	5 d	TNF-α, IL-6, IL-1β, IL-10	MAPK, NF-κB	Sun et al. (2023)
			*In Vitro*	RAW 264.7 cell	NA	20 μM	20 μM	NA	24 h	ERK, p-ERK, p38, p-p38, JNK, p-JNK, p65, p-p65, TNF-α, IL-1β, TGF-β, IL-10	MAPK, NF-κB	Sun et al. (2023)
Theaflavin-3′-gallate	*Camellia sinensis*	Polyphenol	*In Vivo*	BALB/c mice	Female	40 mg/kg/day	40 mg/kg/day	Oseltamivir	7 d	IL-6, IL-10, IL-1β, TLR4, p38, p-p38	MAPK	Sima et al. (2023)
			*In Vitro*	MDCK cell, A549 cell	NA	2.5–40 μg/mL	2.5 μg/mL	Oseltamivir	24–36 h	Influenza virus nucleoprotein	MAPK	Sima et al. (2023)
Baicalin	*Scutellaria baicalensis*	Flavonoid	*In Vivo*	SD rats	Male	10,20,40 mg/kg/day	10 mg/kg/day	NA	7 d	TNF-α, IL-1β, IL-6, P38mapk, p-p38MAPK	MAPK	
Grape seed proanthocyanidin	*Vitis vinifera*	Proanthocyanidin	*In Vitro*	A549 cell, HEp-2 cell	NA	5–10 μg/mL	5 μg/mL	NA	72 h	MUC1, MUC2, MUC5AC, MUC5B, MUC8, ERK1/2, p-ERK1/2, JNK, p-JNK, p38, p-p38, c-Fos, c-Jun, IκBα, p-IκBα, NF-κB p65, p-NF-κB p65	MAPK, NF-κB	Lee et al. (2017)
Phillyrin	*Forsythia suspensa*	Lignan	*In Vivo*	BALB/c mice	Male	15 mg/kg/day	15 mg/kg/day	Oseltamivir phosphate	7 d	IL-1β, IL-18, CCL11, CCL3, CCL5, CCL2, CCL3/MIP-1α, CXCL1, NLRP3, Caspase1, Cleaved Caspase1	NLRP3	Zhang et al. (2023)
			*In Vitro*	MDCK cell	NA	25, 50, 100 µM	25 µM	Ribavirin	12–36 h	IL-1β, IL-18, CCL11, CCL3, CCL5, CCL2, CCL3/MIP-1α, CXCL1, NLRP3, Caspase1, Cleaved Caspase1	NLRP3	Zhang et al. (2023)
Cyanidin-3-O-glucoside, Peonidin-3-O-glucoside	*Black rice germ and bran*	Anthocyanin	*In Vitro*	A549 lung cell, THP-1 macrophage	NA	5–20 μM	5 μM	Dexamethasone	24 h	IL-6, IL-1β, IL-18, NF-κB p65, NLRP3, Caspase-1, ASC	NLRP3, NF-κB	Semmarath et al. (2022)
Astragalus polysaccharide	*Astragalus membranaceus*	Polysaccharide	*In Vivo*	Wistar rats	Male and female	50–100 mg/kg/day	50 mg/kg/day	NA	5 d	IL-1β, IL-18, TNF-α, IL-6, NLRP3, Caspase-1, Cleaved Caspase-1, ASC, ROS, SOD, GSH, MDA, Nrf2, HO-1	NLRP3	Shen et al. (2021)
Hesperetin	*Clerodendrum petasites S.Moore*	Flavanone	*In Vitro*	A549 lung cell, THP-1 cell	NA	0–40 μg/mL	5 μg/mL	NA	27 h	IL-6, IL-1β, IL-18, NLRP3, ASC, Caspase-1, Cleaved Caspase-1	Akt/MAPK, NLRP3	Arjsri et al. (2022)

Notes: *In vivo* studies are expressed in mg/kg/day or μg/mL, depending on the route of administration. *In vitro* studies use μM or μg/mL, depending on compound solubility and reporting standards. “NR” indicates data not reported in the original publication, while “NA” means not applicable, such as when sex is irrelevant in cell-based assays.

## 2 NF-κB signalling pathway

### 2.1 The ignition switch of the cytokine storm

The NF-κB signaling pathway is a key regulatory network in the body’s inflammatory and antiviral immune responses. Under steady-state conditions, NF-κB (such as the p50/p65 heterodimer) is bound to the inhibitory protein inhibitor of kappa B (IκB) and remains inactive (Hayden and Ghosh, 2004). After viral invasion, pathogen-associated molecular patterns (PAMPs) activate the IκB kinase (IKK) complex through pattern recognition receptors such as toll-like receptors (TLRs) and retinoic acid-inducible gene I (RIG-I), inducing IκB phosphorylation and degradation, and releasing NF-κB into the nucleus to initiate the expression of pro-inflammatory factors such as TNF-α, IL-6, and IL-1β(Yoneyama et al., 2004; Kawai and Akira, 2007). Additionally, NF-κB also induces chemokines, such as C-X-C motif chemokine ligand 8 (CXCL8), and ROS-generating enzymes, such as reduced nicotinamide adenine dinucleotide phosphate (NADPH) Oxidase 2, establishing a positive feedback loop of inflammation amplification with ROS: ROS, in turn, activates IKK and NF-κB transcriptional activity, further enhancing pro-inflammatory signals and forming a “inflammation-oxygen co-stimulation” state (Morgan and Liu, 2011; Liu et al., 2017). In viral pneumonia, the excessive activation of the NF-κB pathway is a key factor in triggering cytokine storms and lung tissue damage. For instance, severe acute respiratory syndrome coronavirus 2(SARS-CoV-2) binds to angiotensin-converting enzyme 2 and induces ROS bursts, continuously activating NF-κB, which causes alveolar epithelial cell apoptosis, increased vascular permeability, and massive infiltration of immune cells, thereby exacerbating pulmonary edema and increasing the risk of acute respiratory distress syndrome (ARDS) (Zhou et al., 2024). Meanwhile, NF-κB can also inhibit antioxidant pathways such as Nrf2, leading to a decline in ROS clearance capacity and forming a vicious cycle (Wardyn et al., 2015). Therefore, precisely targeting the NF-κB pathway or regulating its interaction network with ROS has become an important strategy for alleviating the inflammatory pathology of viral pneumonia.

### 2.2 Natural compounds targeting NF-κB

Natural products have established the central role of NF-κB in the pathogenesis of viral pneumonia and have shown synergistic anti-inflammatory and antiviral effects. Artemisinin-derived artesunate (ART) significantly reduces the expression of TNF-α, IL-6, and IL-1β in the lung tissue of influenza-infected mice by inhibiting the TLR4/NF-κB (p65) axis, with a lung index reduction of over 30%, and significantly improves body weight and survival rate (Zhou et al., 2022). Patchouli alcohol (PA) blocks NF-κB activation by up-regulating the negative regulatory protein ubiquitin-specific peptidase 18 and simultaneously inhibits NLRP3 inflammasome and gasdermin D-mediated pyroptosis, increasing the survival rate of hemagglutinin type 1 and neuraminidase type 1 influenza A virus (H1N1)-infected mice by approximately 40% (Jiang et al., 2025). Andrographolide specifically modifies the Cys62 residue of the NF-κB p50 subunit, inhibits its DNA binding activity, and significantly reduces the phosphorylation level of p65 (Ser536) (Western blot shows a 65% decrease in p-p65), reducing inflammatory cell infiltration in lung tissue (Ding et al., 2017). Quercetin-3-O-rutinoside inhibits the phosphorylation of IκBα and p65, significantly inhibits the expression of TNF-α, IL-6, and interferon-stimulated gene 54 in human alveolar epithelial cells (A549) and reduces the ratio of p-IκBα/IκBα and p-p65/p65 in the lung tissue of infected mice, with a nearly 2-log reduction in viral load and a reduction in lesion area of over 50% (Luo et al., 2023). Moreover, β-Escin not only shows dose-dependent viral load inhibition in SARS-CoV-2 and canine coronavirus infection models but also inhibits the NF-κB/IL-6/TNF-α signaling axis in macrophages, significantly reducing the levels of inflammatory factors, demonstrating broad-spectrum antiviral and immunomodulatory activities (Peñaranda Figueredo et al., 2024). Overall, these natural products can effectively alleviate the immune pathological responses caused by viruses by targeting key nodes of NF-κB, regulating ROS interactions, or jointly blocking downstream inflammatory networks, providing diverse and stable candidate options for the adjuvant treatment of viral pneumonia.

## 3 PI3K/akt signalling pathway

### 3.1 The metabolic–inflammatory hub

The PI3K/Akt signaling pathway is one of the core pathways mediating cell survival, metabolism, and immune regulation. Its activation state is often hijacked during viral infection to facilitate viral replication and immune evasion. Multiple viruses, including influenza virus, syncytial virus (RSV), and SARS-CoV-2, can activate the PI3K/Akt pathway to inhibit the pro-apoptotic protein B-cell lymphoma 2 (Bcl-2)-associated agonist of cell death, enhance Bcl-2 expression, thereby delaying host cell apoptosis and extending the viral replication window (Diehl and Schaal, 2013). Additionally, Akt activation can upregulate Programmed Death-Ligand 1 expression, inhibit interferon signaling and the transcription of antiviral genes, weaken T cell-mediated immune clearance capacity, and enhance viral immune evasion (Jorgovanovic et al., 2020). Furthermore, Akt can further activate the downstream mechanistic target of rapamycin (mTOR) pathway, promoting host cell protein synthesis and energy metabolism, thereby providing resource support for the viral life cycle (Basile et al., 2022). In viral pneumonia, PI3K/Akt activation also participates in inflammatory regulation: on one hand, it promotes NF-κB activation by phosphorylating IκB, inducing the production of inflammatory factors such as IL-6 and TNF-α, which can easily trigger cytokine storms and ARDS; on the other hand, this pathway participates in the M1/M2 phenotype conversion of macrophages and the regulation of the TLR/mTOR axis, affecting immune metabolic reprogramming and tissue repair in the lungs (Zhao et al., 2014). In summary, the PI3K/Akt pathway exhibits highly complex “double-edged sword” characteristics in viral pneumonia, serving as both a metabolic/immune platform exploited by viruses and a potential target for host regulation of inflammation and repair.

### 3.2 Natural compounds targeting PI3K/Akt

Based on the pathological role of the PI3K/Akt pathway in viral pneumonia, many naturally active products have been found to exert synergistic antiviral and anti-inflammatory effects by negatively regulating this pathway. Phillyrin significantly inhibits p-PI3K, p-Akt, and NLRP3 expression in respiratory RSV-infected mice, upregulates the antioxidant factor Nrf2, reduces IL-6 and IL-1β levels in lung tissue, and decreases viral mRNA expression by approximately 35% (Wang et al., 2024). Rosmarinic acid shows similar effects, reducing RSV-F gene expression and lung index, and improving pathological damage (Wang et al., 2024). Resveratrol induces the expression of p-Akt and Caspase-3, synergistically promoting infected cells to enter a programmed apoptotic state, thereby controlling inflammation and viral spread (Li et al., 2020). Decursin significantly reduces viral load in the lungs of influenza-infected models (>1 log), downregulates the ratio of p-PI3K/PI3K and p-Akt/Akt, and regulates the balance of IL-6, IL-17, TNF-α, and IL-10, effectively alleviating morphological abnormalities in lung tissue (Tang et al., 2025). PA not only downregulates the phosphorylation of PI3K/Akt and extracellular signal-regulated kinase (ERK)/MAPK pathways but also reduces viral load in lung tissue of influenza A virus (IAV)-infected mice by 1.5 log units, improves pathological scores, and increases survival rate to 80%, with overall efficacy comparable to oseltamivir (Yu et al., 2019). In summary, these natural products achieve multi-level comprehensive intervention in viral pneumonia models, from inhibiting viral replication and alleviating the release of inflammatory factors to regulating oxidative stress and tissue protection, by synergistically inhibiting PI3K/Akt and its cross-signaling axes, such as Nrf2, NF-κB, NLRP3, establishing a natural drug action mechanism centered on “integrated regulation of signaling pathways”, providing theoretical support for the subsequent development of multi-target anti-inflammatory and antiviral drugs.

## 4 Nrf2 signalling pathway

### 4.1 The oxidative-stress buffer

Nrf2 is a core transcription factor for cells to respond to oxidative stress. Under normal circumstances, it is maintained at a low expression level through the degradation mediated by the kelch-like ECH-associated protein 1 (Keap1)-Cullin 3-RING-box protein 1 E3 ubiquitin ligase complex (Yamamoto et al., 2018). When ROS, electrophilic metabolites or certain drugs modify the key cysteine residues of Keap1, Nrf2 is released from the complex, translocates into the nucleus and binds to small Maf proteins, recognizing antioxidant response element (ARE) to initiate the expression of downstream antioxidant and detoxification genes such as heme oxygenase-1(HO-1), NADPH quinone dehydrogenase 1 (NQO1), glutamate-cysteine ligase catalytic subunit (GCLC), and superoxide dismutase (SOD), thereby maintaining cellular redox homeostasis (de Freitas Silva et al., 2018; Ngo and Duennwald, 2022). In viral pneumonia, oxidative stress and the inflammatory cascade constitute a key pathological axis. Viruses such as IAV, RSV, and SARS-CoV-2 consume glutathione in large quantities during replication, activate NADPH oxidase and induce a sharp increase in ROS; at the same time, viral components activate the TLR-MAPK-NF-κB pathway, amplifying the release of pro-inflammatory factors such as IL-6 and TNF-α, causing alveolar damage and widespread inflammatory responses (Labarrere and Kassab, 2022; Kayesh et al., 2025). The Nrf2 pathway can intervene in this process at two levels: first, by enhancing the expression of antioxidant enzymes, removing ROS, and restoring the reduced glutathione/oxidized glutathione ratio, blocking the positive feedback of oxidation-inflammation; second, by competing with NF-κB and interferon regulatory factor 3 for co-activator CREB-binding protein, inhibiting the histone acetylation of pro-inflammatory gene promoters, and reducing cytokine expression at the epigenetic level (Harvey et al., 2009; Gao et al., 2021). Animal experiments have confirmed that enhancing Nrf2 activity can significantly improve pulmonary oxygenation function, inhibit exudation reactions, and reduce viral titers, suggesting its important regulatory potential in alleviating oxidative inflammatory damage and promoting tissue repair in viral pneumonia (Cho et al., 2009; Waqas et al., 2023).

### 4.2 Natural compounds targeting Nrf2

Many natural products have been proven to exert synergistic antiviral, antioxidant, and anti-inflammatory effects in viral pneumonia models by activating the Nrf2 signaling pathway. They generally disrupt the Keap1-Nrf2 complex, promote Nrf2 nuclear translocation, and upregulate the expression of antioxidant genes such as HO-1, NQO1, and GCLC, thereby enhancing glutathione synthesis, removing ROS, and alleviating oxidative stress; at the same time, this signaling axis can also jointly inhibit inflammatory pathways such as NF-κB and MAPK, reducing inflammatory damage to lung tissue (Cho et al., 2009; Chaopreecha et al., 2025). In the SARS-CoV-2 infection model, andrographolide significantly inhibited viral replication in African green monkey kidney epithelial cells Vero E6 and human airway epithelial cells at doses of 1.25–10 μM by promoting Nrf2 nuclear translocation and enhancing GCLC expression. At a dose of 1.25 μM, it showed comparable efficacy to 5 μM remdesivir (Chaopreecha et al., 2025). Oral administration of 20 mg/kg/day for 5 days in BALB/c mice (BALB/c) also significantly improved lung inflammation and tissue damage. Similarly, R-sulforaphane upregulates the expression of HO-1 and NQO1 in RSV-infected ICR mice (9 μmol per mouse, 7 days), reduces granulocyte infiltration and viral titers, and activates the Nrf2-ARE axis while jointly regulating the activator protein 1 (AP-1) and NF-κB pathways, demonstrating dual antioxidant and immunomodulatory effects (Cho et al., 2009). Berberine can also inhibit RSV-F expression and IL-6 and TNF-α levels in BALB/c mice by activating the Nrf2-glutathione peroxidase 4 ferroptosis inhibitory axis, and the efficacy of the high-dose group is close to that of ribavirin by gavage at 30–60 mg/kg for 4 days (Gao et al., 2024). Arctiin, when treated at concentrations of 100–300 μg/mL in A549 cells for 48 h, can reverse the downregulation of Nrf2/HO-1 and SOD2 induced by H9N2 virus and block the RIG-I/MAPK pathway. Its anti-inflammatory effect is also dependent on Nrf2 signaling regulation, and the HO-1 inhibitor Zinc Protoporphyrin IX can partially weaken its protective effect (Zhou et al., 2021). Additionally, emodin also shows antiviral and tissue-protective effects dependent on the Nrf2 pathway in the H1N1 virus infection model. At a concentration of 6.25 μg/mL *in vitro*, it can inhibit viral replication, and at doses of 25–75 mg/kg/day for 6 days *in vivo*, it can significantly activate Nrf2, simultaneously inhibit TLR4/p38 mitogen-activated protein kinase (p38)/c-Jun N-terminal kinase signaling pathway (JNK) and NF-κB signaling, improve oxidative stress indicators, reduce the expression of inflammatory factors, and increase the survival rate of mice (Dai et al., 2017). In summary, although these natural products differ in structural origin and target regulation, they generally activate the Nrf2 pathway in various virus infection models to integrate the regulation of oxidative stress, inflammatory response, and viral replication, forming a common mechanism.

## 5 MAPK signalling pathway

### 5.1 The stress amplifier

The MAPK signaling pathway is a conserved cellular cascade that plays a crucial role in regulating cell proliferation, differentiation, stress responses, and immune modulation (Cargnello and Roux, 2011). Upon viral infection, host pattern recognition receptors such as TLRs and RIG-I rapidly activate MAPK cascades to initiate immune responses. Among MAPK subtypes, JNK and p38 are most closely associated with inflammation (Cheng et al., 2024). They phosphorylate c-Jun and activate stress-related proteins, thereby modulating the expression of pro-inflammatory cytokines such as IL-6 and TNF-α, and influencing immune cell polarization and migration. However, excessive activation of MAPK pathways by viral infections can lead to dysregulated cytokine release, disruption of alveolar epithelial and vascular endothelial barriers, pulmonary edema, and impaired gas exchange (Zhou et al., 2024). In SARS-CoV-2 infection, aberrant MAPK activation, partly triggered by viral proteins such as the Envelope protein and Open Reading Frame 7a via ionic imbalance and ROS induction, is regarded as a key driver of cytokine storm and ARDS (Planès et al., 2022; Cheng et al., 2024). Both animal and clinical studies have shown a strong correlation between JNK/p38 activity and pulmonary inflammation markers (Su et al., 2010; Siegl and Uhlig, 2012; Malekinejad et al., 2022). Furthermore, MAPK inhibitors such as SB203580, targeting p38, and U0126, targeting mitogen-activated protein kinase kinase 1 (MEK1)/ERK, have demonstrated anti-inflammatory and tissue-protective effects in various viral pneumonia models, highlighting the MAPK pathway as a promising therapeutic target linking viral recognition to immune pathology (Soares-Silva et al., 2016; Braicu et al., 2019).

### 5.2 Natural compounds targeting MAPK

Building on this pathological basis, multiple natural compounds have been validated to exert dual anti-inflammatory and antiviral effects in viral pneumonia models by modulating key nodes within the MAPK pathway. Notably, natural agents such as kaempferol, theaflavin-3′-gallate (TF2b), baicalin, and grape seed proanthocyanidins (GSP) have been shown to suppress phosphorylation of p38 and JNK, thereby attenuating downstream activation of NF-κB and AP-1 pathways and reducing pro-inflammatory cytokine production (Sima et al., 2023; Sun et al., 2023). Kaempferol promotes M2 macrophage polarization and suppresses JNK transcriptional activity in lipopolysaccharide-induced cytokine storm models, significantly improving mouse survival rates (Sun et al., 2023). TF2b mitigates H1N1-induced lung inflammation by inhibiting the TLR4/MAPK/p38 axis and downregulating viral nucleoprotein expression (Sima et al., 2023). Baicalin reduces phosphorylated p38 MAPK levels by approximately 45% and restores CD3^+^/CD8^+^ T cell ratios, enhancing immune clearance of RSV. GSP exhibits broad regulatory effects by blocking both p38 and JNK phosphorylation, inhibiting AP-1 and NF-κB (p65/IκBα) signaling, and dose-dependently reducing viral titers by 60%–75% at 5–10 μg/mL (Lee et al., 2017). Collectively, these findings indicate that the MAPK pathway serves not only as a central amplifier of post-infection inflammation but also as a key molecular target for natural product-based modulation of immune pathogenesis in viral pneumonia. The observed differences in subtype selectivity, immunomodulatory reprogramming, and effects on viral replication stages offer insights for structure-based precision intervention.

## 6 NLRP3 inflammasome

### 6.1 Dual-signal activation and pyroptosis

The NLRP3 inflammasome, composed of NLRP3, apoptosis-associated speck-like protein containing a CARD (ASC), and pro-caspase-1, serves as a critical innate immune sensor that mediates pro-inflammatory cytokine release in response to various PAMPs and damage-associated molecular patterns (Kelley et al., 2019). Its activation follows a two-signal model: Signal 1 is initiated by viral nucleic acid recognition via receptors such as TLRs and RIG-I, leading to NF-κB activation and transcriptional upregulation of NLRP3 and pro-IL-1β; Signal 2 involves cellular perturbations including Potassium ion efflux, mitochondrial ROS accumulation, and lysosomal rupture, which trigger inflammasome assembly and caspase-1 activation (Li et al., 2019; Effendi and Nagano, 2021). This subsequently leads to the cleavage of IL-1β/IL-18 and gasdermin D-mediated pyroptosis (Kelley et al., 2019). Potassium ion efflux, mitochondrial ROS(mtROS)-induced oxidized mitochondrial DNA (mtDNA) release, and lysosomal cathepsin leakage are regarded as the three core events for NLRP3 activation (Ravi Kumar et al., 2018; Freeman and Swartz, 2020). In viral pneumonia, NLRP3 functions dually as a mediator of antiviral defense and a driver of immunopathology (Li et al., 2024). For instance, during early influenza infection, TLR7/RIG-I signaling and M2 ion channel activity cooperatively promote potassium ion efflux and mtROS generation, activating NLRP3 to enhance IL-1β/IL-18 release (Ichinohe et al., 2010; Effendi and Nagano, 2021). However, excessive activation during late infection stages contributes to cytokine storms and ARDS. Similarly, in COVID-19, SARS-CoV-2 structural proteins activate NLRP3 through ion imbalance, ROS induction, or direct inflammasome engagement. Downstream IL-1β/IL-18 promote amplification of IL-6 and TNF-α signaling, neutrophil extracellular trap formation, and microthrombi, all of which contribute to ARDS pathogenesis (Kaivola et al., 2021; Vora et al., 2021). Thus, therapeutically targeting NLRP3 or its cytokine cascade holds promise for alleviating severe inflammation, though its clinical application requires precise temporal control to balance immune defense and tissue protection (Freeman and Swartz, 2020).

### 6.2 Natural compounds targeting NLRP3

A growing body of research has focused on the anti-inflammatory and antioxidant effects of natural compounds targeting various stages of NLRP3 activation during viral infections. For example, berberine, a classic isoquinoline alkaloid, effectively inhibited mitochondrial ROS generation and mitochondrial antiviral signaling protein activation in influenza-stimulated in influenza-stimulated mouse macrophage cell line J774A.1 (J774A.1), thereby blocking NLRP3 assembly and pyroptosis within 24 h at 4.2 μM(Zhao et al., 2025). Phillyrin demonstrated a multitargeted inhibitory effect in BALB/c mice and Madin-Darby canine kidney (MDCK) cells, suppressing both the NLRP3/caspase-1 axis and the CXCR2 chemotactic pathway. At doses of 15 mg/kg·day or 25 μM, it showed comparable efficacy to oseltamivir and ribavirin, with therapeutic onset within 7 days or 36 h (Zhang et al., 2023). In SARS-CoV-2 models, anthocyanins such as cyanidin-3-glucoside (Cy-3-G) and peonidin-3-glucoside (Pn-3-G) from black rice germ extracts suppressed spike protein subunit 1 protein-induced NLRP3 assembly, ASC oligomerization, and caspase-1 activation in A549 and human monocytic leukemia cell line (THP-1), reducing IL-1β/IL-18 secretion within a 5–20 μM range, similar to dexamethasone (Semmarath et al., 2022). Moreover, some compounds modulate upstream transcription factors to attenuate NLRP3 expression. Rhein reduced NLRP3 levels by inhibiting NF-κB activation in human bronchial epithelial cell line BEAS-2B (BEAS-2B) and mice, while astragalus polysaccharides downregulated NLRP3, caspase-1, IL-1β, and IL-18 after 5-day treatment at 50 mg/kg in H1N1-infected rats (Shen et al., 2019; Shen et al., 2021). In the coronavirus-related model, hesperetin effectively reduced the mRNA and protein expression of IL-6, IL-1β and IL-18 by blocking the interaction network between Akt/MAPK/AP-1 and NLRP3 in A549 and THP-1 cells, with a minimum active concentration of 5 μg/mL and an action time of 27 h, suggesting that it has dual effects of cytoprotection and inflammation antagonism (Arjsri et al., 2022). These natural molecules can synergistically block the initiation and cascade of NLRP3 inflammasome through multi-target synergistic blocking in viral pneumonia models, providing a solid experimental basis for the development of adjuvant therapy strategies with broad-spectrum immunomodulatory effects.

## 7 Synthesis and future perspectives

TCM natural products in treating viral pneumonia lie in their “multi-target, multi-pathway” synergistic regulation of the inflammation-oxidative stress network, establishing a dynamic balance of “anti-inflammatory, antioxidant, and antiviral” effects (Zhang et al., 2025). These compounds primarily act on key signaling nodes, including NF-κB, Nrf2, PI3K/Akt, MAPK, and the NLRP3 inflammasome. To further elucidate the crosstalk mechanisms, several studies have identified shared molecular switches that integrate these pathways. For example, IKKβ not only mediates NF-κB activation but also interacts with Keap1, a redox-sensitive regulator of Nrf2, thereby linking oxidative and inflammatory responses. PI3K/Akt signaling promotes Nrf2 nuclear translocation and antioxidant gene expression, while reducing intracellular ROS and indirectly suppressing both NF-κB and NLRP3 activation. Natural products such as curcumin inhibit IKKβ and covalently modify cysteine residues on Keap1, thus activating Nrf2 and blocking inflammatory transcription (Rahban et al., 2020). Similarly, berberine activates PI3K/Akt signaling, stabilizes Nrf2, and concurrently downregulates NF-κB activity (Zhang et al., 2016). These findings exemplify how natural compounds can coordinate multi-pathway regulation through critical signal convergence hubs, achieving redox–immune homeostasis. Despite structural diversity, compounds from different chemical classes—including flavonoids, polyphenols, terpenoids, alkaloids, anthraquinones, lignans, and polysaccharides—often converge mechanistically (Daskou et al., 2023; Guo et al., 2024). For instance, flavonoids (e.g., isoquercitrin, kaempferol, baicalin, hesperetin) and polyphenols (e.g., rosmarinic acid, resveratrol, theaflavin-3′-gallate, proanthocyanidins) consistently suppress NF-κB-mediated cytokine production and activate Nrf2 signaling, often via their antioxidant and free radical-scavenging properties (Lee et al., 2017; Li et al., 2020; Arjsri et al., 2022; Luo et al., 2023; Shen et al., 2023; Sima et al., 2023; Sun et al., 2023; Wang et al., 2024). Terpenoids (e.g., andrographolide, patchouli alcohol, artesunate) regulate multiple inflammatory axes—including TLR4/NF-κB, PI3K/Akt, JAK/STAT, and Nrf2—to modulate both immune activation and apoptosis (Ding et al., 2017; Yu et al., 2019; Zhou et al., 2022). Alkaloids like berberine, and anthraquinones like emodin, inhibit both NF-κB and MAPK signaling while modulating caspase-mediated apoptosis (Dai et al., 2017; Zhao et al., 2025). Lignans (e.g., phillyrin) and polysaccharides (e.g., astragalus polysaccharide) simultaneously target NLRP3, NF-κB, and Nrf2 to restore immune–redox balance (Zhang et al., 2023). These functional effects are closely associated with the compounds’ structural features. Shared pharmacophores—such as α,β-unsaturated carbonyls, hydroxyl-rich aromatic rings, and quaternary ammonium groups—facilitate simultaneous interactions with multiple inflammatory and oxidative signaling proteins (Bousquet et al., 2020; Fu et al., 2022). Moreover, physicochemical parameters like hydrophilicity, molecular size, and charge influence bioavailability, tissue distribution, and phase-specific efficacy. For example, hydrophilic polysaccharides and glycosylated flavonoids accumulate in alveolar fluid and are suited for mucosal inflammation, while lipophilic terpenoids and anthraquinones cross membranes more rapidly to target viral replication compartments or redox-sensitive proteins (Huang et al., 2022). This structure–function correspondence forms the mechanistic foundation for multi-target optimization and synergistic combination strategies in natural product–based antiviral drug development. However, even compounds sharing identical pharmacophores may exhibit divergent signaling behaviors due to differences in stereoelectronic properties and molecular frameworks. For example, both artesunate and andrographolide contain α,β-unsaturated carbonyl groups, yet their distinct backbone rigidity, hydrophobicity, and functional moieties confer different pathway preferences. Artesunate, with its endoperoxide bridge and potent ROS-modulating capacity, predominantly suppresses the NF-κB signaling pathway (Wang et al., 2017). In contrast, andrographolide, owing to its conjugated structure and electrophilicity, tends to modulate the MAPK cascade. In TNF-α–stimulated cells, andrographolide significantly inhibits the phosphorylation of p38 MAPK and ERK1/2, supporting its anti-inflammatory mechanism of action (Li et al., 2017). While these findings demonstrate robust regulatory activity across multiple pathways, key unresolved questions remain regarding their upstream specificity, timing, and viral context. Despite substantial progress in delineating the roles of NF-κB, Nrf2, PI3K/Akt, MAPK, and NLRP3 pathways, several mechanistic gaps remain. For example, the specific upstream regulators of distinct MAPK subtypes in viral pneumonia remain poorly defined. Similarly, the temporal and context-dependent dual roles of PI3K/Akt, which can support viral replication but also mediate anti-inflammatory signaling, warrant further investigation. Furthermore, the precise timing and regulation of NLRP3 inflammasome activation in balancing immune defense versus tissue injury is still not fully understood. Future studies should address these pathway-specific gaps to optimize therapeutic interventions.

In addition, the physicochemical characteristics of different classes of natural compounds—including hydrophilicity, lipophilicity, molecular size, and charge—substantially influence their tissue distribution and stage-specific therapeutic efficacy. Hydrophilic polysaccharides and glycosylated flavonoids diffuse efficiently into alveolar fluid, making them particularly suited for mitigating early-phase mucosal inflammation at the macrophage–epithelial interface. In contrast, lipophilic terpenoids and anthraquinones more readily penetrate cell membranes and accumulate in viral replication compartments or redox-sensitive targets. There, they may exert antiviral or anti-inflammatory effects by inducing ferroptosis or covalently modifying key regulators such as Keap1 and IKKβ via peroxide bridges or Michael addition (Orosco and Quimque, 2024; Guo et al., 2021; Kiser et al., 2021; Das et al., 2023; Zhao et al., 2024). Typically, small molecules act rapidly to scavenge ROS and suppress early inflammation, whereas macromolecules or saponins bearing sugar moieties tend to accumulate in immune cells and exert prolonged anti-cytokine effects (Jen et al., 2021; Shao et al., 2022; Shen et al., 2024). While structure–function regularities support the multitarget potential of natural products, their therapeutic effects are shaped by the specific biological context of each viral infection. Therefore, effective application of TCM-derived compounds in viral pneumonia requires careful consideration of pathogen-specific immune and oxidative response profiles. For instance, SARS-CoV-2 strongly activates the NLRP3 inflammasome, while H1N1 influenza virus preferentially stimulates NF-κB and MAPK cascades (Wang and Li, 2023). These mechanistic differences may underlie the virus-specific efficacy of certain natural compounds. Baicalin has been shown to inhibit NLRP3 activation and alleviate lung injury in SARS-CoV-2 models (Wang and Li, 2023). In contrast, resveratrol, a known NF-κB inhibitor, exhibits greater anti-inflammatory activity in H1N1-induced lung inflammation (Rossi et al., 2021). In addition, differences in viral entry mechanisms may influence how effectively natural compounds reach and modulate early intracellular signaling targets (Caffrey and Lavie, 2021; Jackson et al., 2022). These variations highlight the need for mechanistic validation in multiple viral infection models when assessing the therapeutic potential of multitarget agents. Building upon these mechanistic and physicochemical insights, it is also crucial to compare the broader pharmacological frameworks of natural products versus conventional Western medicines, especially in the context of antiviral therapy. Western antiviral agents such as oseltamivir and remdesivir exert rapid viral suppression by targeting specific viral proteins, with the advantages of well-defined mechanisms and direct onset of action. However, their clinical application remains challenged by narrow target specificity, the risk of resistance development, and limited capacity to modulate host-driven inflammatory responses. In contrast, natural products exhibit multi-target and holistic regulatory properties, showing potential in alleviating host inflammation and immune dysregulation associated with viral infections. Furthermore, recent studies have demonstrated the synergistic potential of combining natural products with Western medicines. For instance, baicalin has been reported to enhance the therapeutic efficacy of oseltamivir in influenza-infected mice by reducing proinflammatory cytokines, alleviating pulmonary inflammation, and improving survival outcome (Ding et al., 2014). Similarly, multi-component formulations such as Lianhua Qingwen have also been reported to potentiate the antiviral and anti-inflammatory effects of oseltamivir in influenza models, further supporting the rationale for integrative therapy approaches (Yang et al., 2020). These findings suggest that integrative treatment strategies may provide superior outcomes by simultaneously targeting viral replication and host immune dysregulation. As such, the inclusion of natural products in antiviral combination regimens represents a promising direction for clinical translation and personalized therapy.

Currently, natural products from TCM demonstrate multi-target synergistic advantages in regulating inflammation and oxidative stress to treat viral pneumonia, but their practical application still faces significant limitations. First, the development and industrialization of natural products are hindered by complex extraction and purification processes. The stability and consistency of bioactive compounds—such as alkaloids and saponins—are susceptible to variations in herbal source, harvesting season, and processing methods, resulting in batch-to-batch fluctuations and impeding standardization of formulation and quality control (Leong et al., 2020; Guo et al., 2025). Second, significant bottlenecks exist in the clinical translation of mechanistic studies. Most investigations are restricted to animal models or immortalized cell lines, which often fail to recapitulate the physiological behavior of primary human alveolar epithelial cells due to altered receptor expression, metabolic pathways, and redox dynamics. This limits the translatability of *in vitro* findings (Ma et al., 2024). Moreover, substantial species-specific differences in immune signaling between rodents and humans, including immune cell distribution, cytokine profiles, and kinase activity, further compromise the predictive value of animal studies. These cross-species discrepancies challenge both efficacy and toxicity extrapolation to clinical settings. To address these issues, future research should prioritize the use of human-relevant models, such as patient-derived lung organoids, and cross-species comparative transcriptomics (Leach et al., 2020; Tindle et al., 2021; Han et al., 2022). These platforms may provide more accurate insights into therapeutic mechanisms and safety profiles, thereby facilitating clinical translation of TCM-based interventions. Additionally, the potential toxicity of long-term administration on metabolic organs such as the liver and kidneys remains insufficiently evaluated. Moreover, few studies have systematically addressed the dose–effect–toxicity relationship, which is essential for defining the clinical safety profile of natural products. Many compounds exhibit narrow therapeutic windows, and increasing the dose beyond the effective range may lead to hepatic or renal toxicity (Kim et al., 2012). Notably, the effective dose for modulating inflammatory responses may differ from that required for direct antiviral effects, suggesting the need for dual-endpoint dose optimization. To enhance both safety and clinical translatability, future investigations should integrate pharmacokinetic and pharmacodynamic data, such as maximum plasma concentration, area under the concentration-time curve, and tissue distribution profiles, to precisely define the therapeutic window and optimize exposure levels. Such integrative strategies may help mitigate dose-related organ toxicities, particularly during long-term administration (Rombolà et al., 2020; Zagaliotis et al., 2022). Finally, current studies on the target mechanisms of natural medicines remain focused on common antiviral or inflammatory pathways, with limited exploration of virus-specific host interaction nodes (Chen and Ye, 2022).

Future research should prioritize the following directions to improve the translational feasibility of natural product–based therapies: On the one hand, integration of systems biology and artificial intelligence should be leveraged to construct multi-layered “component–target–pathway–disease” networks via network pharmacology. This would help elucidate spatiotemporal regulatory patterns of multi-component formulations on inflammation–oxidative stress axes, with functional validation in organoid or lung-on-chip models to better simulate tissue-level repair. On the other hand, clinical research must be deepened and refined. Large-scale, multi-center randomized controlled trials should be designed to evaluate the long-term impacts of natural products on pulmonary lesion resolution and organ function. These assessments can integrate metabolomics, advanced imaging, and biomarker-based stratification to support precision interventions. Importantly, formulation innovation should be grounded in the physicochemical limitations of natural products. For example, lipophilic terpenoids with poor membrane permeability may benefit from self-emulsifying drug delivery systems or solid lipid nanoparticles, while rapidly cleared polysaccharides may require chitosan-coated nanocarriers or polymeric conjugation to improve stability and bioavailability (Jörgensen et al., 2020; Guadarrama-Escobar et al., 2023; Kumar et al., 2025; Uttreja et al., 2025). Concurrently, synthetic biology can be employed to optimize the biosynthetic pathways for producing high-purity active ingredients under standardized quality control frameworks (Nasim et al., 2022). By integrating the holistic principles of TCM with modern precision medicine and pharmaceutical technology, it may be possible to overcome key bottlenecks in the clinical translation of natural medicines for viral pneumonia.

## Glossary

AP-1: Activator Protein 1
ARDS: Acute respiratory distress syndrome
ARE: Antioxidant response element
ART: Artesunate
ASC: Apoptosis-associated speck-like protein containing a CARD
BALB/c: A common laboratory mouse strain
Bcl-2: B-cell lymphoma 2
BEAS-2B: Bronchial Epithelial Cells
Cov: Coronavirus
COVID-19: Coronavirus Disease 2019
CXCL: C-X-C Motif Chemokine Ligand
Cy-3-G: Cyanidin-3-glucoside
DNA: Deoxyribonucleic Acid
ERK: Extracellular signal-regulated kinase
GSP: Grape seed proanthocyanidins
H1N1: Hemagglutinin Type 1 and Neuraminidase Type 1 Influenza A Virus
HO-1: Heme Oxygenase-1
IAV: Influenza A Virus
IKK: IκB kinase
IL-6: Interleukin-6
IRF3: Interferon Regulatory Factor 3
IκB: Inhibitor of kappa B
J774A.1: A murine macrophage cell line
JUK: C-Jun N-terminal Kinase signaling pathway
Keap1: Kelch-like ECH-associated protein 1
MAPK: Mitogen-Activated Protein Kinase
MDCK: Madin-Darby Canine Kidney cells
mtDNA: Mitochondrial DNA
mTOR: Mechanistic target of rapamycin
mtROS: Mitochondrial ROS
NADPH: Reduced Nicotinamide Adenine Dinucleotide Phosphate
NF-κB: Nuclear Factor kappa-light-chain-enhancer of activated B cells
NLRP3: NOD-like receptor family pyrin domain containing 3
NOD1: NAD(P)H Quinone Dehydrogenase 1
Nrf2: Nuclear factor erythroid 2–related factor 2
P38: P38 Mitogen-Activated Protein Kinase
p65: RelA (part of NF-κB)
PA: Patchouli alcohol
PAMPs: Pathogen-associated molecular patterns
PI3K/Akt: Phosphoinositide 3-kinase/Protein Kinase B
Pn-3-G: Peonidin-3-glucoside
RIG: Retinoic acid-Inducible Gene
RNA: Ribonucleic Acid
RNS: Reactive nitrogen species
ROS: reactive oxygen species
RSV: Respiratory syncytial virus
SARS: Severe Acute Respiratory Syndrome
TCM: Traditional Chinese Medicine
TF2b: Theaflavin-3′-gallate
THP-1: Human monocytic leukemia cell line
TLRs: Toll-Like Receptors
TNF-α: Tumor necrosis factor-alpha
